# TNF-α as a Therapeutic Target in Acute Pancreatitis — Lessons from Experimental Models

**DOI:** 10.1100/tsw.2007.98

**Published:** 2007-03-30

**Authors:** Giuseppe Malleo, Emanuela Mazzon, Ajith K. Siriwardena, Salvatore Cuzzocrea

**Affiliations:** ^1^ Department of Clinical, Experimental Medicine and Pharmacology, School of Medicine, University of Messina, Italy; ^2^ IRCCS Centro Neurolesi “Bonino-Pulejo”, Messina, Italy; ^3^ Department of Surgery, Hepatobiliary Unit, Manchester Royal Infirmary, Manchester, UK

**Keywords:** cytokines, experimental pancreatitis, anti-TNF-α therapy, clinical trial

## Abstract

A considerable body of experimental evidence suggests that tumor necrosis factor (TNF)-α plays a major role in several aspects of inflammation and shock. In particular, it is pivotal in many detrimental effects of acute pancreatitis, and it represents a major determinant of the systemic progression and end-organ damage (such as acute lung injury and liver failure) of this pathologic condition. Given the importance of TNF-α in the pathogenesis of acute pancreatitis, investigators have regarded blocking the action of this mediator as an attractive treatment option. Different specific and nonspecific inhibitors have been developed with promising results in animal models, but, on the other hand, no clinical trials have been designed so far. Difficulties in clinical applications may be multifactorial; experimental models are not fully reliable and reproduce at least some aspects of human disease, timing of intervention should be related to changes in TNF-α serum levels, and inclusion criteria should be accurately selected to better define the population most likely to benefit.

## INTRODUCTION

Acute pancreatitis is a common disease with great variability in severity. Whereas it runs a fairly benign course in most patients, in others it can take a severe form characterized by extensive necrosis and in-hospital mortality rates in excess of 25%[1]. It has been demonstrated that many individuals facing severe pancreatitis develop dysfunction of a mean of two organs, indicating that multiple organ failure (MOF) occurs. Overall, the main causes of death include circulatory shock; cardiac insufficiency; and renal, respiratory, and hepatic failure[2]. Therefore, the prognosis of severe acute pancreatitis is highly dependent on appropriate measures to prevent MOF, and much effort has been done to improve understanding of the mechanisms of disease progression from acinar cell injury to an overwhelming, life-threatening illness.

Once proteolytic enzymes become activated and pancreatic tissue digested, patients show a similar pattern of aggravation, characterized by an exaggerated inflammatory response. A relevant aspect of inflammation, which in the last decade catalyzed the research on acute pancreatitis, is the overproduction of soluble mediators, such as cytokines. These molecules are up-regulated from the early stages of the disease and are directly correlated to many deleterious events locally in the pancreas as well as in distant organs[3].

Tumor necrosis factor (TNF)-α is the prototypic member of a cytokine family that mediates a broad spectrum of responses to stress and injury. It is primarily produced by immune cells, such as monocytes and macrophages, but many other cell types are also capable of its release, including acinar cells. The biologic effects of TNF-α are mediated by two distinct surface receptors, namely TNF-α receptor 1 (TNFR1), or p55, and TNF-α receptor 2 (TNFR2), or p75[4].

A primary role for TNF-α in sepsis, endotoxemic shock, and acute pancreatitis is suggested by several observations[2,5,6]. During pancreatitis, TNF-α overproduction is pivotal in the induction of inflammatory genes, cell death, endothelial up-regulation, and in the recruitment and activation of immune cells[7]. Accordingly, the source, the kinetic, the regulation, and the effects of TNF-α have been considered as critical points in the comprehension of the temporal course and of the sequences of pathophysiological changes; and investigators have regarded blocking the production or the action of this cytokine as an attractive treatment option.

Owing to the rapid onset of human pancreatitis, current knowledge on the role of TNF-α as a major mediator in the development of the disease and as a potential therapeutic target mainly springs from studies conducted on animal models[8]. Growing evidence showed that TNF-α blockade attenuates the development of experimental pancreatitis, but – on the other hand – no clinical studies were conducted due to difficulties in translational research and the disappointing results obtained in sepsis trials.

This review outlines the recent findings on the involvement of TNF-α in experimental acute pancreatitis, evaluating points that may influence the design of future clinical trials on TNF-α neutralization.

## MATERIALS AND METHODS

A computerized MEDLINE search was made to identify articles regarding the role of TNF-α in acute pancreatitis. The MeSH headings “pancreatitis” and “tumor necrosis factor-alpha” yielded, respectively, 33,126 and 55,218 hits. These two major headings were combined to produce 287 papers. Then the single headings and the meshed result “pancreatitis AND tumor necrosis factor-alpha” were variously combined to restrict the search with the keywords “experimental model”, “TNF receptors”, “cytokines”, “apoptosis”, “neutrophils”, “macrophages”, “oxidative stress”, and “endothelium”. After that, 145 relevant papers were selected, retrieved, and studied in full text. We included reviews and original contributions; single case reports and non-English papers were excluded. A further manual review of the reference list of the articles selected was made and papers missed by electronic searching were retrieved in full manuscript.

## AN OVERVIEW ON EXPERIMENTAL MODELS OF ACUTE PANCREATITIS

As randomized studies in the clinical setting have inherent limitations, experimental models of acute pancreatitis are of particular relevance for investigating the pathophysiology of the disease, and for evaluating potential new pharmacological agents or other therapeutic strategies. Rodents (e.g., mice and rats) are the most widely used animals. Current models reproduce the cardinal features of human pancreatitis (elevation in serum levels of amylase/lipase, histological changes, and pancreatitis-associated complications) and gene-targeting techniques provide an additional focus on specific, key biological factors involved in the pathogenesis[8,9].

The methods of pancreatitis induction can be invasive or noninvasive, ranging from simple diet to exogenous chemical administration or even surgical manipulation. On the basis of the induction techniques, experimental models of pancreatitis can be divided into five categories.

## 1. Secretagogue-Induced Experimental Acute Pancreatitis

Secretagogue-induced pancreatitis is based on the introduction (generally via the intraperitoneal route) of agents that increase proteolytic enzyme secretion, causing pancreatic acinar autolysis. These models are easy to undertake, noninvasive, and highly reproducible. The two major agents used are cerulein and L-arginine. Secreatagogue-induced pancreatitis provides an excellent approach to study early acinar pathophysiological events; the principal disadvantage is the deficient clinical relevance.

Cerulein is a cholecystokinin-pancreozymin analogue. Supramaximal doses cause interstitial pancreatitis, sharing many features with acinar histological changes seen in human disease[9].

The basic amino-acid L-arginine produces selective acinar cell necrosis and adipose tissue necrosis around the pancreas; the temporal course of pathologic changes of pancreatitis (patterns of disease and recovery) mirrors that observed in the clinical setting[10].

## 2. Diet-Induced Experimental Acute Pancreatitis

A choline-deficient, ethionine supplemented (CDE) diet produces severe necrotizing pancreatitis in young female mice. The consequence of this diet is depletion of S-adenosylmethionine, which causes impairment in pancreatic homeostasis with extensive necrosis, edema, and inflammatory infiltration. Pancreatitis severity can be controlled by limiting the diet feeding period. Gross histological appearance of the pancreas resembles the human disease; hypoxia, hypovolemia, acidosis, and ascites occur[10].

The disadvantages of this model are that the amount of injury depends critically on the gender and age of the mice, and dietary intake must be constant between groups.

## 3. Duct Injection-Induced Experimental Acute Pancreatitis

Duct injection-induced experimental acute pancreatitis is a surgical model that requires laparotomy and cannulation of the pancreatic duct. Injection of sodium glycodeoxycholate (a bile salt secretagogue) and perfusion of active pancreatic enzymes produces an acute edematous pancreatitis, while retrograde injection of sodium taurocholate in male rats produces a severe and rapidly worsening cellular injury[10].

The development of acute pancreatitis is followed by a mortality rate of 5–10% and the histologic injury looks similar to those in human disease (edema, leukocyte infiltration, parenchymal hemorrhage, and necrosis). The main limitations are complexity, invasiveness, and expense.

## 4. Duct Ligation-Induced Experimental Acute Pancreatitis

Duct ligation-induced experimental acute pancreatitis is performed by placing a ligature around the common bile duct (CBD), producing an early pancreatitis with obstructive jaundice. Avoidance of the use of drugs that may have unspecified side effects and the parallels with acute biliary pancreatitis with biliopancreatic reflux are the key strengths of this model[10].

Several surgical variants of the duct ligation model have been described using duodenal loops with gastroduodenostomy in addition to the CBD ligation. The mechanism of induction of pancreatitis seems to be related to duodenopancreatic reflux. Coexisting duodenal necrosis and peritoneal sepsis often complicate the model, making the evaluation of the results complicated.

## 5. Microvascular Injury-Induced Experimental Acute Pancreatitis

In microvascular injury-induced models, pancreatitis is caused by ischemic injury after occluding pancreatic vessels. Polystyrene microspheres and arterial ligation can be used. A popular model is the ischemia/reperfusion-induced acute pancreatitis. Oxygen free radicals, activation of leukocytes, failure of microvascular perfusion, cellular acidosis, and disturbance of intracellular homeostasis appear to be important factors in the pathophysiology of the ischemia/reperfusion-mediated injury[10]. Such mechanisms are thought to produce pancreatic injury in the clinical setting, especially after major surgery, coagulopathy, and microvascular thrombosis.

## PATHOPHYSIOLOGY OF TNF-A IN EXPERIMENTAL PANCREATITIS

## TNF-α and Early Events in Acinar Cells

Several experimental studies demonstrated that during the early phases of acute pancreatitis, TNF-α is primarily up-regulated within the pancreas. Norman and colleagues first showed intrapancreatic TNF-α gene expression, resulting in large amounts of protein product with levels consistently higher than those found in the serum[11]. In addition, Denham et al. demonstrated that preventing the activity of TNF-α by TNFR1 genetic deletion has beneficial effects on the severity and mortality in cerulein-induced pancreatitis[12].

In another study by Gukovskaya et al., acinar cells were shown to release and respond to TNF-α, and it was therefore postulated that the increased intrapancreatic levels of TNF-α and TNF receptors (TNFR1/TNFR2) could also be due to acinar production, but the relative contribution of each cell type (acinar cells vs. leukocytes) in the protein bioactivity could not be ascertained[13]. Subsequently, the principal effort has been directed toward identifying the actual source of this mediator at a cellular level, although activated immune cells, such as monocytes and macrophages, infiltrating the pancreas have been considered the most prominent source of TNF-α, accruing evidence shifted the focus to acinar cells[14]. Comparative *in vivo* kinetic studies evaluated the time-course of TNF-α production in experimental pancreatitis by semi-quantitative analysis of mRNA expression[15] or, more recently, by flow cytometry[16,17], showing that this cytokine is up-regulated first in acinar cells and later in immune cells infiltrating the pancreas[15].

The present results indicate that acinar cells submitted to stress promote the inflammatory process, not only releasing active enzymes locally and lipids systemically, but, more surprisingly, behaving like "real" inflammatory cells. This emerging concept has been very recently reinforced by the observation of acinar TNF-α production after challenge with pancreatitis-associated ascitic fluid (PAAF)[18], and by the intra-acinar finding of the leukocyte-specific receptor-like protein CD45[19,20]. CD45 levels inversely correlate with the production of TNF-α and this molecule may act as a negative regulator of pancreatic inflammatory response.

TNF-α exerts its proinflammatory actions mainly through the activation of different genes, including those for other cytokines, chemokynes, cell adhesion molecules, cyclooxygenase-2 (COX-2), and inducible nitric oxide synthase (iNOS). The encoding of inflammation-related genes is triggered by signaling cascades involving protein kinases and transcriptional factors, such as nuclear factor (NF)-kB, STAT, and the steroid hormone family peroxisome proliferators–activated receptors (PPARs)[9,21][Fig. 1].

TNF-α is a strong inductor of NF-kB, and we have proven that interfering in NF-kB pathway is an effective strategy to decrease the inflammatory response during experimental pancreatitis[22,23]. We have also demonstrated cross-talk between tyrosine kinase–mediated cellular signaling and the systemic release of TNF-α[24], as well as the role of PPAR-α and PPAR-γ in attenuating TNF-α production and pancreatic inflammation[9,25].

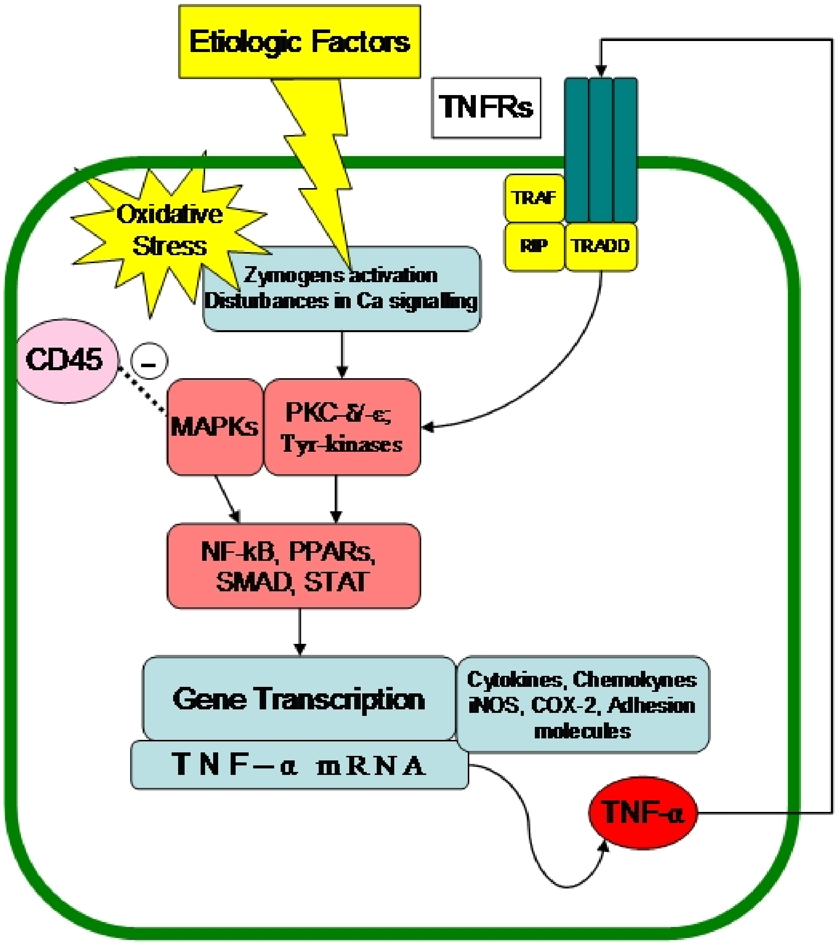

**FIGURE 1**. Model of TNF-α production in acinar cells during pancreatitis. A kinases cascade triggered by disturbances in cell homeostasis activates a number of transcription factors, such as NF-kB, thereby leading to TNF-α up-regulation. CD45, formerly a leukocyte-specific glycoprotein, may have a role in negative regulation of TNF-α production through MAPK silencing. An overproduction of TNF-α enhances the inflammatory response via interactions with its specific receptors and recruitment of different adaptor proteins. Tyr-Kinases = tyrosine kinases; MAPK = mitogen activated protein kinases; TNFRs = TNF receptors; NF-kB = nuclear factor kB; PPARs = peroxisome proliferator–activated receptors; TRAF = TNF receptor–associated factor; TRADD = TNF receptor–associated death domain; RIP = receptor interacting protein.

In particular, tyrosine kinase inhibitor treatment influences the phosphorylation of mitogen activated protein kinases (MAPK), a family of serine/threonine kinases that includes three subfamilies: extracellular signal-regulated kinase (erk 1/2), c-Jun $NH_2$-terminal protein kinase (JNK), and p38. Their activation in the pancreas by extracellular signals and stressful stimuli (e.g., secretagogues and cytokines) mediates different cellular events, including TNF-α expression[26]. MAPK selective inhibition was associated with TNF-α down-regulation and resulted in a significant amelioration of experimental acute pancreatitis[27,28].

Apart from its effects on gene transcription, TNF-α has also been demonstrated to cause cytoskeletal disorganization through activation of proline-rich tyrosine kinase 2 (pyk2) and to trigger cell death signaling through different pathways mediated by the specific protein-kinase C isoforms PKC-δ and PKC-ε[29].

In conclusion, ongoing research strongly suggests that in the early stages of experimental pancreatitis, acinar cells are the primary source of TNF-α as well as of other pro- and anti-inflammatory mediators.

Therefore, the extent, progression, and severity of the disease may initially depend on the relative imbalance between pro- and anti-inflammatory responses at an acinar level. The subsequent up-regulation of proinflammatory genes and the secretion of protein products may constitute the first signal to attract leukocytes to the damaged pancreas.

## From Acinar Cells to a Systemic Response

In response to proinflammatory stimuli arising from acinar cells, an intricate sequence of events involving the tissue vasculature and inflammatory cells occurs. TNF-α intensifies oxidative stress by converting xanthine dehydrogenase to xanthine oxidase[30] and, together with other cytokines and reactive oxygen species (ROS), it causes relevant alterations in capillary endothelial cells and postcapillary venules, which lose their selective permeability and become procoagulant and proadhesive. An uncontrolled up-regulation of adhesion molecules, such as selectins and ICAM-1, promotes rolling, adhesion, aggregation, and transmigration of leukocytes into inflamed tissues[31]. Excessively stimulated leukocytes play an important role in both pancreatic and systemic manifestation of acute pancreatitis through the secretion of secondary mediators including TNF-α itself.

### *Neutrophils*

Neutrophils are recruited from the general circulation, where their levels are increased from the early stages in various models of pancreatitis[32]. These cells are the major contributor of tissue injury through the activation of iNOS (NADPH oxidase-dependent mechanism)[33]. In our laboratory, we showed that the absence of iNOS, as well as the administration of superoxide dismutase mimetics, significantly reduces the development of cerulein-induced pancreatitis in mice[34,35].

Furthermore, neutrophils infiltrating the pancreas have also been recently shown to contribute via ROS to a further pathologic activation of digestive enzymes in the pancreas as well as to the activation of nuclear enzyme poly (ADP-ribose) polymerase (PARP) through strand breaks in DNA[31]. Growing evidence suggests that PARP-1 inhibition modulates the inflammatory process and negatively regulates TNF-α and other proinflammatory cytokines; we and others recently demonstrated that TNF-α levels in the pancreas were markedly reduced in mice treated with 3-aminobenzamide, a PARP-1 inhibitor[36,37]. Moreover, TNF-α directly enhances chemotaxis and activation of neutrophils, resulting in further superoxide production. This important contribution of neutrophils to necrotic cell death has been confirmed by the significant increase in acinar cells undergoing apoptosis observed using antineutrophil serum[38]. The progression of necrosis and the late apoptotic acinar cell death seems influenced by the local presence of neutrophils via a TNF-α–dependent mechanism.

Accruing experimental evidence suggests that neutrophil infiltration plays a pivotal role in the progression of acute lung injury, which is indistinguishable from adult respiratory distress syndrome (ARDS)[39]. This is a common, early complication of acute pancreatitis and is a major cause of early mortality in severe disease. Recently, Lundberg and colleagues reported a correlation between TNF-α release, up-regulation of pulmonary ICAM-1/VCAM-1, neutrophils sequestration, and lung injury in experimental pancreatitis[40].

As showed in Fig. 2, neutrophils and oxidative stress, together with TNF-α and adhesion molecules, coact in complex cross-talk and generate a vicious circle that amplifies the inflammatory response and accounts for tissue damage.

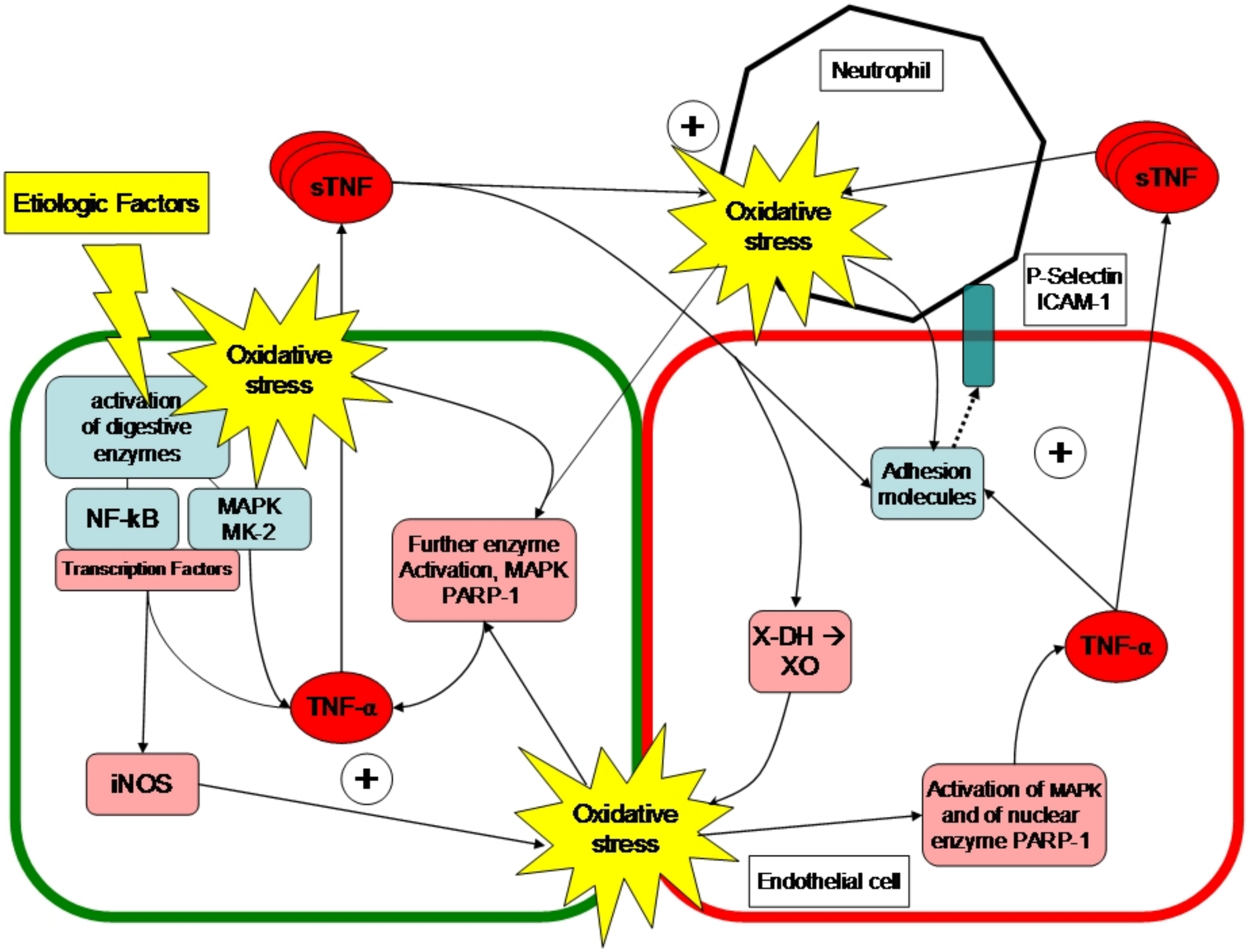

**FIGURE 2.** The vicious circle generated by TNF-α, adhesion molecules, neutrophils, and oxidative stress. ROS are initially produced in acinar cells, endothelial cells, and neutrophils. TNF-α up-regulates the expression of adhesion molecules, activates neutrophils, and converts xanthine dehydrogenase into xanthine oxidase in endothelial cells. Oxidative stress enhances the activation of the nuclear enzyme PARP-1 and of MAPK. PARP-1 and MAPK account for further production of TNF-α. sTNF = soluble TNF-α; NF-kB = nuclear factor kB; iNOS = inducible nitric oxide synthase; ROS = reactive oxygen species; MAPK = mitogen activated protein kinases; MK-2 = mitogen activated protein kinases activated protein kinase 2; PARP-1 = poly(ADP-ribose) polymerase; X-DH = xanthine dehydrogenase; XO = xanthine oxidase.

## *Monocytes and Macrophages*

Circulating monocytes and resident macrophages play a primary role in the development of systemic inflammatory response syndrome (SIRS) during acute pancreatitis[41,42]. Activated peripheral blood monocytes are able to produce spontaneously large amounts of TNF-α[43], and their systemic up-regulation seems triggered by early signals released from the pancreas that gain access to the general circulation. Specific pancreatic enzymes, such as elastase, carboxypeptidase A, and lipase, were shown to activate and induce TNF-α expression in a rat macrophage cell line[44], although some recent reports pointed out that pancreatic elastase free of contamination by endotoxins fails to induce murine macrophages to release the cytokine[45].

Peritoneal mononuclear cells, which are involved in the defense of the abdominal cavity against infection, are responsible for excessive production of TNF-α in pancreatitis[46]. It is known that PAAF may modulate the function of peritoneal macrophages, resulting in augmented TNF-α levels[47], and that experimental necrotizing pancreatitis is characterized by a marked decrease of peritoneal macrophages, probably due to tissutal homing or necrotic death.

Another mechanism activating macrophages may derive from their physiologic scavenging activity. Necrotic debris, which behaves like pathogen-associated molecular pattern (PAMPs), is recognized by toll-like receptors (TLR), such as TLR 4 and TLR 2[48].

The largest resident macrophage population is the hepatic Kupffer cell mass. It has been widely demonstrated that pancreatic elastase induces exaggerated production of TNF-α, whose augmented levels contribute to pancreatitis-associated liver injury (in a NF-kB- and MAPK-dependent fashion) and to hepatocyte apoptosis[49,50]. Furthermore, chemotactic mediators from the liver, including TNF-α, seem to play a crucial role in the activation of alveolar macrophages and consequently in the development of neutrophil infiltration and lung injury[51]. Therefore, the ability to manipulate peripheral blood monocytes and resident macrophages may influence the systemic inflammatory response and may have important therapeutic implications.

### *Circulating TNF-α and Role of Soluble TNF Receptors*

Although different studies have shown a prolonged increase in TNF-α serum levels in experimental pancreatitis[9,11,21,22,23,24,25,33,34,35], there are conflicting reports indicating that TNF-α concentrations are elevated, in a transient fashion, only in the initial stages of the disease[52]. This may be interpreted as a consequence of the interfering effect of soluble TNF-α receptors.

Soluble TNF-α receptors (sTNFR) directly derive from proteolytic cleavage of membrane TNFR1 and TNFR2, they bind to circulating free TNF-α, and their shedding from cell membrane is markedly enhanced soon after the onset of experimental pancreatitis[53]. Granell and colleagues, by using two different assays that discriminate between the free form of TNF-α and that bind to proteins, observed that the cytokine is mainly present bound to specific proteins. The concomitant increased concentrations of sTNFR strongly suggested that they could be these binding proteins[53].

To date, the biologic significance of sTNFR is still unclear, as their physiological neutralizing capacity seems relatively low (so that, in theory, a 30- to 300-fold molar excess would be necessary to buffer the bioactivity of circulating TNF-α, they may serve as TNF-α carriers into the circulation). The fact that elevated concentrations of sTNFR in the absence of detectable concentrations of free TNF-α were associated to distant organ injury supports the view that sTNFR may play a proinflammatory role[52].

In contrast, it was recently reported by Alsfasser and colleagues that pancreatic elastase may account for circulating TNF-α inactivation. The authors conclude that it is unlikely that TNF-α plays an important role in systemic manifestations of experimental pancreatitis[54].

### *Conclusions*

TNF-α and other cytokines are produced within the pancreas as well as systemically during experimental acute pancreatitis, and have been implicated in the progression of the inflammatory process. This hypothesis is supported by the fact that inhibitors of cytokines tend to ameliorate the associated distant organ dysfunctions. Published evidence supporting a direct role of TNF-α in experimental pancreatitis is summarized in Table 1.

## TNF-α and "Apoptosis vs. Necrosis" in Experimental Pancreatitis

Both necrosis and apoptosis occur in experimental pancreatitis. It is now well known that the severity of the disease is related to the type and the degree of cell death induced by different etiologic factors; severe pancreatitis is associated with extensive acinar cell necrosis, while mild pancreatitis reveals extensive apoptotic cell death and a minimal amount of necrosis[55]. Thus, apoptosis has been interpreted as a beneficial cell response to injury, and inducing apoptosis is an effective strategy to decrease the severity of experimental pancreatitis[56].

**TABLE 1**
**Direct Evidence for a Central Role of TNF-α in the Pathogenesis of Experimental Acute Pancreatitis**

| Experimental Model | Observations | Ref. |
|---|---|---|
| **Pancreas level** | | |
| Cerulein AP | TNF-α gene expression is induced within the pancreas during AP, resulting in large amounts of protein product with levels consistently higher than those found in the serum. | 11 |
| | Preventing the activity of TNF-α by genetic deletion of TNFR1 has beneficial effects on the severity and mortality of AP. | 12 |
| | Pancreatic acinar cells produce, release, and respond to TNF-α after supramaximal stimulation with cerulean. | 13 |
| | Pancreatic acinar cells submitted to stress activate TNF-α gene expression, with maximal protein production at 6 h after cerulein administration. | 15 |
| BPDO-induced AP | Acinar cells produce TNF-α with a maximum at 6–12 h after the induction of AP, releasing the protein in the peripheral blood earlier than inflammatory cells. | 16 |
| | PAAF activates the production of TNF-α in acinar cells. | 18 |
| Isolated rat pancreatic acini and AR42J cells | TNF-α causes acinar cell cytoskeletal disorganization and NF-kB activation, triggering also cell death signaling via PKC-δ/-ε. | 29 |
| Cerulein AP | TNFR1 knockout mice are protected against biochemical and histological manifestation of AP. | 72 |
| **Distant sites** | | |
| Cerulein AP | Subsequent to the early intrapancreatic formation, TNF-α is produced in large amounts in lung, liver, and spleen. | 11 |
| CDE AP | AP is associated with TNF-α systemic release followed by increased expression of pulmonary adhesion molecules, neutrophil infiltration, and lung injury. | 40 |
| Taurocholate AP | Peritoneal inflammatory cells (macrophages, neutrophils) increase after the induction of AP, with substantially raised TNF-α levels in the lavage fluid. | 46 |
| BPDO-induced AP | Circulating monocytes are activated early in the course of AP and produce large amounts of TNF-α. | 43 |
| NR8383 MΦ cell line | Specific pancreatic enzymes activate MΦ to produce TNF-α via NF-kB. | 44 |
| Rat liver perfused with pancreatic elastase | Pancreatic elastase induces liver injury by activating TNF-α production and gene expression within Kupffer cells via NF-kB. | 50 |
| Taurocholate AP | sTNFR are released in the early stages of experimental AP and this increase is concomitant with the release of TNF-α, which is mainly bound to specific proteins. | 53 |
| | Significant increase in serum levels of TNF-α and sTNFR is observed after the induction of AP. | 52 |

AP = Acute pancreatitis; TNFR1 = TNF receptor-1; BPDO = bile-pancreatic duct obstruction; PAAF = pancreatitis-associated ascitic fluid; NF-kB = nuclear factor-kB; PKC = protein kinase C; CDE = choline deficient-ethionine supplemented; MΦ = macrophages; sTNFR = soluble TNF receptors.

However, the mechanisms through which acinar cells fall into apoptosis or necrosis are still unclear. Different regulating events, a delicate balance of mediators and of pro- and antiapoptotic members of the bcl-2 gene family acts in an intricate network that finally determines the pattern of cell death[57,58]. Cellular energy status could be a critical factor regulating the apoptosis/necrosis ratio (as apoptosis requires ATP for activating caspases), and the overactivation of the nuclear enzyme PARP, which causes ATP depletion[36], has been regarded as a cardinal event in inducing necrosis.

TNF-α is capable of stimulating acinar cell death through necrosis and/or apoptosis[13]. By means of interactions with TNFR1, it can activate signaling complexes leading to the apoptotic arm or to NF-kB/MAPKs pathways[59][Fig. 3]. These events are influenced at various levels, including regulation of receptor/ligand expression and antiapoptotic molecules induction.

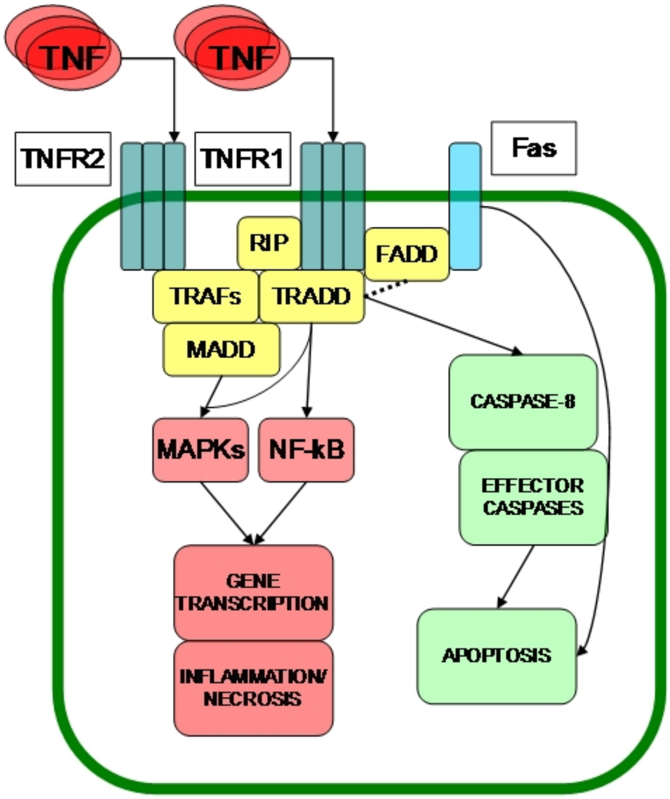

**FIGURE 3**. TNF-α mediates both apoptosis and necrosis. TNF-α can trigger both apoptosis and inflammation/necrosis through recruitment of different adaptor proteins. TRADD is the proximal transducer of apoptosis and requires an interaction with FADD to activate caspase-8. The inflammatory effects of TNF-α are mediated by an intricate association of TRADD, TRAFs, RIP, and MADD that "switch on" gene transcription mainly through NF-kB and MAPKs. TNFR1/2 = TNF-α receptors 1/2; TRAFs = TNF receptor–associated factors; RIP = receptor interacting protein; TRADD = TNF receptor–associated death domain; FADD = Fas-associated death domain; MADD = mitogen activated kinases death domain; NF-kB = nuclear factor kB; MAPKs = mitogen associated protein kinases.

One key regulator of TNF-α–induced cell death is NF-kB that – apart from its role in promoting the inflammatory response – has been shown to down-regulate apoptosis. NF-kB neutralization results in a marked potentiation of caspases activity, which inhibits necrosis and intra-acinar enzyme activation[60]. The antiapoptotic effects of NF-kB activation involve the expression of cellular inhibitors of apoptosis (IAP-1/2, XIAP), which inactivate downstream caspases, and of the caspase-8 inhibitor FLIP[61].

Another candidate for mediating antiapoptotic effects is pancreatitis-associated protein I (PAP I), that has been demonstrated to induce a significant apoptosis decrease in rat acinar cells exposed to TNF-α[62].

Recent findings have pointed out that TNF-α–mediated signaling can also promote an alternative and coordinated form of nonaccidental necrosis that sets apart from the "classic" necrosis triggered by cellular stress and ATP depletion[63]. This newly discovered cell death mechanism has been called "programmed necrosis" or "necrosis-like programmed cell death". To date, the only proven mediator of this alternative pathway is the adaptor protein RIP, whose deficiency protects against TNF-α–induced programmed necrosis. Mareninova and colleagues showed that RIP degradation by caspases correlates with low necrosis and high apoptosis degree, demonstrating that RIP signals programmed necrosis and that caspases may exert a protective effect in experimental pancreatitis[64].

In conclusion, to determine cell death pathways in pancreatitis and to identify strategies to switch necrosis to apoptosis may have a therapeutic value. Besides its well-defined proinflammatory activities in the early stages of the disease, TNF-α mediates proapoptotic effects (especially when NF-kB is blocked) as well as programmed necrosis. Given the central role of TNF-α in determining cell death, further studies are required to better clarify the intracellular events involved and the consequences of therapeutic strategies on cytokines inhibition.

## TNF-α AS A THERAPEUTIC TARGET IN ACUTE PANCREATITIS

There are still no specific therapies for acute pancreatitis and treatment remains largely supportive, although in the last few years studies have shown a reduction in mortality in patients receiving prophylactic antibiotics[65] and also in individuals undergoing endoscopic sphincterotomy for severe gallstone-related disease[66].

In view of the central role of TNF-α in the innate host inflammatory response, investigators have regarded blocking the production or the action of this cytokine as an attractive treatment option for a variety of conditions associated with excessive or poorly controlled inflammation. Several strategies have been developed for neutralizing TNF-α, including polyclonal antibodies[67], monoclonal antibodies[68,69,70], soluble receptors constructs[71,72], and nonspecific agents (e.g., thalidomide, phosphodiesterase inhibitors, metalloproteinase inhibitors, and others)[73,74,75,76,77,78,79,80,81,82, 83,84][Table 2]. In the last decade, more than 140 preclinical studies have been conducted to assess the effects of TNF-α neutralization in models of acute infection or inflammation (especially sepsis) and to identify appropriate patient populations for therapeutic intervention[85]. Subsequently, 12 completed phase II and III randomized clinical trials have been carried out in human sepsis, showing only a very modest impact on mortality, although in a highly heterogeneous population of patients[86,87,88].

Few experimental studies and no clinical trials have been conducted on TNF-α neutralization in acute pancreatitis so far. Selective inhibitors of cytokine production have been reported to prevent histological changes and to improve the survival rate in closed duodenal loop pancreatitis in rats[89]. Denham and colleagues – by administering an inhibitor of macrophage production of TNF-α and IL-1 – showed a dramatic reduction in tissue damage and in pancreatitis severity in mice[90]. Another anticytokine agent with rheologic properties that significantly reduced histological score, biochemical manifestations, and glutathione depletion in different experimental models of pancreatitis was the metylxanthine derivative pentoxifylline[80,81]. The simultaneous inhibition of TNF-α and xanthine oxidase, with pentoxifylline and oxypurinol, respectively, abolished the inflammatory changes associated with taurocholate pancreatitis[91]. Anti-TNF-α antibodies have been initially used to evaluate the temporal relationship between induction of pancreatitis and the rise of TNF-α in serum. In particular, pretreatment with polyclonal anti-TNF-α antibodies in experimental pancreatitis inhibits the early burst of TNF-α activity and significantly improved the course of the disease as well as overall survival in rats, thereby demonstrating that an early selective blockage of TNF-α may be of value[67,92,93]. Very recently, Oruc and colleagues reported that infliximab, a monoclonal anti-TNF-α antibody, ameliorates the course of both edematous and severe necrotizing pancreatitis in rats, although in severe disease, it did not influence mortality rate, neutrophil activity, and pancreatic edema[70].

The first study evaluating the early and delayed effects of a recombinant dimeric form of soluble TNFR1 (p55) was conducted in rat choline-deficient diet pancreatitis. A marked amelioration of biochemical parameters, histological score, lung associated injury, and overall survival were observed either in prophylactic or delayed treatment, whereas mortality rate was significantly diminished in the

**TABLE 2**
**Specific and Nonspecific Pharmacologic Agents Targeting TNF-α**

| Pharmacologic Agents | Mechanism of Action | Ref. |
|---|---|---|
| Specific | | |
| Anti-TNF polyclonal antibody | Neutralization of circulating TNF-α | 67 |
| Anti-TNF m(F(ab')2) (MAK195F) | Neutralization of circulating TNF-α | 68 |
| Rabbit anti-TNF monoclonal antibody | Neutralization of circulating TNF-α | 69 |
| Chimeric anti-TNF monoclonal antibody (Infliximab) | Neutralization of circulating TNF-α | 70 |
| Recombinant pegylated dimeric-sTNFR1 | Neutralization of circulating TNF-α | 71 |
| sTNFR2:Fc dimeric fusion protein (Etanercept) | Neutralization of circulating TNF-α | 72 |
| Nonspecific | | |
| Thalidomide and analogues (CC-3052) | Enhanced TNF mRNA degradation | 73,74 |
| Interleukin-10 | Decreased production of TNF-α | 75 |
| Interleukin-13 | Decreased translation of TNF mRNA | 76 |
| Pyrinidyl imidazoles (SK&F 86002) | Decreased translation of TNF mRNA | 77 |
| Prostacyclin analogues (Iloprost) | cAMP-mediated inhibition of TNF gene transcription | 78 |
| Phosphodiesterase inhibitors (Rolipram, Pentoxifylline) | cAMP-mediated inhibition of TNF gene transcription | 79,80,81 |
| Metalloproteinase inhibitors (Batimastat/BB-94) | Decreased post-transcriptional TNF-α production | 82 |
| Adenosine | Decreased TNF gene transcription | 83 |
| Tyrosine-kinase inhibitors (Tyrphostins AG126/AG556) | Inhibition of TNF-mediated intracellular signaling | 24 |
| Macrophages inhibitor (CNI-1493) | Decreased translation of TNF mRNA | 84 |

latter group[71]. Finally, we have demonstrated for the first time that Etanercept, a recombinant humanized soluble TNFR2 (p75) fusion protein, significantly reduces the development of cerulein-induced pancreatitis in mice. Comparing the results with mice lacking TNFR1, we observed that the entity of the reduction of the inflammatory parameters analyzed was similar in wild-type and knockout mice[72]. These results show that TNF-α may be a novel target by therapeutic applications for treating pancreas inflammation. However, the transition from laboratory to clinical settings is problematic and several factors that may influence future clinical trials in acute pancreatitis have to be discussed.

## DISCUSSION

The hallmark of severe acute pancreatitis is the induction of an inflammatory response. Nearly all patients with even modest pancreatitis will exhibit some systemic symptoms, such as fever, whereas individuals who overproduce inflammatory mediators face life-threatening complications, such as SIRS[57]. Primary end-organ dysfunction may follow; and the infection of pancreatic necrosis, together with bacterial translocation, sepsis, and eventually multiple organ failure, accounts for late mortality, after the second week[66]. Therefore, acute pancreatitis represents a paradigm of sterile inflammation in which the systemic inflammatory response may dictate disease severity and outcome.

TNF-α is considered to be one of the major mediators associated to the systemic tissue damage, and its pathophysiology has been widely investigated in order to develop a new medical treatment strategy for acute pancreatitis. Preclinical studies on TNF-α inhibition showed promising results, but a full extrapolation of experimental data has to be made with caution.

More than 2000 papers on the treatment of acute pancreatitis in experimental models have been published, but only a few of the substances tested have translated from laboratory to clinical practice. Difficulties in clinical applications may be multifactorial; animal models mimic some aspects of human pancreatitis, but overall they fail to reproduce the disease accurately and they are not completely predictive for the pathophysiology or treatment[10].

Furthermore, it has been observed that in human pancreatitis, the majority of organ failure occurs before initiation of treatment, and novel agents that ameliorate the course of experimental pancreatitis may be less effective when administered in human trials several hours or days after the onset of disease[94]. There is also the need to use widely accepted prognostic indices to categorize the disease severity and to select inclusion criteria and interpretable end-points.

TNF-α biology *in vivo* is complex. Although TNF-α neutralization attenuates the systemic inflammatory response, it does so at the cost of impairing innate antimicrobial defenses, especially against intracellular pathogens. Furthermore, opportunistic infections and hepatotoxicity were reported[85]. Thus, anti-TNF-α therapy could be even harmful in those conditions, such as sepsis, in which microbial growth contributes the disease pathogenesis. On the contrary, blocking TNF-α could be useful in conditions where microbial proliferation does not occur, and this is in accordance with the results obtained neutralizing TNF-α in experimental models of endotoxiemia[95]. Acute pancreatitis may represent a suitable disease for TNF-α antagonism, being – especially in the early phases – a condition in which the systemic inflammatory response is not initiated and driven forward by an infection. However, three large clinical trials of TNF-α inhibitors in congestive heart failure (a clinical condition of noninfectious origin) were prematurely halted for lack of benefit or adverse outcomes (including increased mortality)[96,97]. In addition, meta-analyses of anti-TNF-α trials in patients with rheumatoid arthritis demonstrate a significant dose-dependent risk of infections and malignancies[98].

What, then, are the factors that may influence a future trial on anti-TNF-α therapy in pancreatitis? It seems critical that the temporal course of events, such as changes in serum TNF-α levels, is related to trial intervention. On one hand, an early TNF-α neutralization could be able at least to decrease the cytokine burst and the induction of further cytokines production by inflammatory cells, but – as seen – in clinical settings, it may not be easy to observe patients within 1 or 2 h from the onset of the disease, when TNF-α peaks in the circulation. Therefore, a potential trial needs to record the time from onset of symptoms to intervention, in addition to the more conventionally recorded delay between hospital admission and intervention[94]. On the other hand, delayed intervention, mostly using TNF-α receptor constructs, has proven to be moderately effective in LPS challenging in human volunteers and septic shock, in a dose-dependent fashion[85]. Although delayed trials would interfere with a multisystemic TNF-α secretion, it must be remembered that the TNF-α responsible for tissue injury may not be the free circulating form, but rather the cell-associated protein. Moreover, at this stage there is an activation of downstream events in different organs, and TNF-α overproduction is only an aspect of that intricate cytokine network that characterizes host response in acute pancreatitis.

In conclusion, what is the lesson we can learn from animal models of pancreatitis and from previous experiences with clinical trials? It should be kept in mind that experimental studies may increase the apparent efficacy of TNF-α antagonism, generating unreasonable expectations as the strategy is translated to clinical settings. Finally, timing of antagonism, and careful selection of inclusion and exclusion criteria may aid in better defining the population most likely to benefit.

## ACKNOWLEDGMENTS

The authors would like to thank Giovanni Pergolizzi and Carmelo La Spada, Mrs. Caterina Cutrona for secretarial assistance, and Miss Valentina Malvagni for editorial assistance with the manuscript.
